# Metformin with Versus without Concomitant Probiotic Therapy in Newly Diagnosed Patients with Type 2 Diabetes or Prediabetes: A Comparative Analysis in Relation to Glycemic Control, Gastrointestinal Side Effects, and Treatment Compliance

**DOI:** 10.5152/tjg.2022.211063

**Published:** 2022-11-01

**Authors:** Kübra Şahin, Yasin Şahintürk, Gökhan Köker, Gülhan Özçelik Köker, Feyzi Bostan, Mehmet Kök, Seyit Uyar, Ayhan Hilmi Çekin

**Affiliations:** 1 Department of Internal Medicine, University of Health Sciences Antalya Training and Research Hospital, Antalya, Turkey

**Keywords:** Type 2 diabetes, metformin, probiotic, BB-12, gastrointestinal intolerance, glycemic control, lipids, body weight, compliance

## Abstract

**Background:**

To evaluate the impact of concomitant use of probiotic BB-12 in metformin-treated patients with type 2 diabetes or prediabetes on glycemic control, metformin-related gastrointestinal side effects, and treatment compliance.

**Methods:**

A total of 156 patients (mean [standard deviation] age: 50.9 [9.9 years], 74.4% females) with newly diagnosed type 2 diabetes or prediabetes were randomly assigned to receive either metformin alone (n = 84, MET group) or metformin plus *Bifidobacterium animalis* subsp. *lactis* (BB-12) probiotic (n = 72, MET-PRO group). Data on body mass index (kg/m^2^), fasting blood glucose (mg/dL), blood lipids, and glycated hemoglobin (HbA1c) levels were recorded at baseline and at the third month of therapy. Data on gastrointestinal intolerance symptoms and treatment noncompliance were also recorded during post-treatment week 1 to week 4.

**Results:**

MET-PRO versus MET therapy was associated with a significantly higher rate of treatment compliance (91.7% vs 71.4%, *P *= .001), greater reduction from baseline HbA1c values (0.9 [0.4-1.6] vs 0.4 [0-1.6] %, *P *< .001) and lower likelihood of gastrointestinal intolerance symptoms, including abdominal pain (*P* = .031 to <.001), diarrhea (*P* = .005 to <.001) and bloating (*P* = .010 to <.001). Non-compliance developed later (at least 15 days after the therapy) in a significantly higher percentage of patients in the MET group (*P *= .001 for 15–21 days and *P *= .002 for 22–28 days).

**Conclusion:**

In conclusion, the present study proposes the benefit of combining probiotics with metformin in the treatment of patients with T2D or prediabetes in terms of improved glycemic control and treatment adherence rather than correction of dyslipidemia or weight reduction.

## Introduction

Given the proposed link between type 2 diabetes (T2D) and altered composition of the gut microbiota, the metabolic potential of the gastrointestinal tract and gut microbiota is increasingly recognized as promising therapeutic targets to improve T2D management.^1,2^

Interventions targeting the gut microbiota (consumption of fibers or direct administration of beneficial bacteria, i.e., probiotics) are suggested to result in significant changes in bacterial composition that are aligned with improvements in glucose control, and experimental studies and clinical trials support the likelihood of this type of modulation of the intestinal microbiota to be effective in diabetes management.^1-5^

The members of the *Bifidobacterium* genus appear to be the most widely studied probiotics with respect to diabetes as consistently reported to be potentially protective against T2D and ameliorate glucose intolerance.^4,6,7^

Recent studies also indicate that the glucose-lowering effects of metformin are mediated by changes in the composition and function of gut microbiota, such as enrichment of microbiota with increased potential to produce short-chain fatty acids (SCFAs), butyrate, and propionate.^1,3,8-10^ Notably, metformin is also considered to impact the relative abundance of several phyla that may contribute to gastrointestinal distress, irrespective of potential impacts on glycemic control, which is the leading cause of metformin intolerance.^1,4^ Undesirable gastrointestinal side effects are considered to affect up to 30% of metformin-treated patients and have been attributed to an increased abundance of *Escherichia* species.^9-11^

Hence, the effects of metformin on gut microbiota are considered responsible not only for its therapeutic effect but also for its undesirable digestive symptoms that may lead to discontinuation or dramatic reduction in daily therapeutic doses, limiting its therapeutic efficacy as a first-line antidiabetic agent.^9-11^

*Bifidobacterium *BB-12 (BB-12) is a catalase-negative, rod-shaped bacterium classified as *Bifidobacterium animalis* subsp. *lactis* and has well-established probiotic characteristics with proven beneficial gastrointestinal and immune health effects.^12^

The impact of probiotics on glucose metabolism has not been extensively studied in patients with the clinical diagnosis of T2D as well as in the setting of ongoing anti-diabetic treatment.^1-3,7,10,11^ Given that probiotics are considered useful in the treatment of gastrointestinal complaints such as diarrhea, bloating, and abdominal pain by rearranging the gastrointestinal microbiota,^10,11^ we have hypothesized that the use of BB-12 in metformin-treated patients may ameliorate the metformin-related side effects alongside their potential additive effects in improved glucose metabolism.

This study was therefore designed to investigate the effects of probiotic supplementation (BB-12) added to metformin treatment on gastrointestinal complaints, compliance with metformin therapy, and glycemic control in patients with newly diagnosed prediabetes or T2D.

## MATERIALS AND Methods

### Study Population

A total of 156 patients (mean [SD] age: 50.9 [9.9 years], 74.4% were females) with newly diagnosed T2D or prediabetes who were planned to receive metformin monotherapy were included in this prospective study conducted between May 2019 and June 2020 at a tertiary care internal medicine clinic. Patients were randomly assigned via a simple randomization method (computer-generated random number sequence) to receive either metformin alone (n = 84, MET group) or metformin plus probiotic support (n = 72, MET-PRO group). Patients with inflammatory bowel disease (IBD), previous metformin therapy, chronic diarrhea, chronic dyspepsia, and diabetic autonomous neuropathy were excluded from the study.

T2D diagnosis was based on the presence of at least one of the following criteria: fasting blood glucose (FBG) ≥ 126 mg/dL, 2h plasma glucose ≥ 200 mg/dL in oral glucose tolerance test (OGTT), HbA1c levels ≥ 6.5% and blood glucose levels ≥ 200 mg/dL at any time in the presence of classical hyperglycemia symptoms. Prediabetes was diagnosed based on the presence of at least one of the following criteria: impaired fasting blood glucose, impaired glucose tolerance, and HbA1c levels of 5.7%–6.4%.

Written informed consent was obtained from each subject following a detailed explanation of the objectives and protocol of the study, which was conducted in accordance with the ethical principles stated in the “Declaration of Helsinki” and approved by the Clinical Research and Ethics Committee of the University of Health Sciences Antalya Training and Research Hospital, Antalya, Turkey (Date of Approval: May 30, 2019; Reference number/Protocol No: 14/13).

### Treatments

All patients received metformin with weekly dose increments (1 × 500 mg, 2 × 500 mg, 3 × 500 mg, and 4 × 500 mg at week 1, week 2, week 3, and week 4, respectively), while those in the MET-PRO group also received daily 4.6 mg *Bifidobacterium animalis* subsp. *lactis *(BB-12) therapy for a month, starting from the first day of metformin therapy.

### Assessments

Data on body mass index (BMI, kg/m^2^), FBG (mg/dL), blood lipids including high-density lipoprotein cholesterol (HDL-C), and low-density lipoprotein cholesterol (LDL-C) and glycated hemoglobin (HbA1c) levels were recorded at baseline and at the third month of treatment. Data on gastrointestinal intolerance symptoms (nausea, vomiting, diarrhea, abdominal pain, bloating, and loss of appetite), taste disturbance, headache, and dizziness were recorded at baseline and post-treatment from week 1 to week 4. Gastrointestinal intolerance symptoms relevant for metformin-related side effects were selected from the abdominal pain, diarrhea syndrome, and indigestion syndrome symptom clusters defined in the Gastrointestinal Symptom Rating Scale (GSRS).^13,14^ Data on treatment noncompliance (treatment discontinuation, dose skipping, or dose reduction) were also recorded. Laboratory parameters and gastrointestinal symptoms were evaluated among patients compliant with treatment in treatment groups (MET vs MET-PRO).

### Statistical Analysis

Statistical analysis was made using IBM SPSS Statistics for Windows, Version 23.0 (IBM Corp., Armonk, NY, USA). Fisher’s exact test and Pearson chi-square analysis were performed for categorical variables. The normality assumptions of the analysis of the two-group measurement differences were controlled by the Shapiro–Wilk test. Mann–Whitney *U* test was used for the analysis of non-normally distributed numerical data while the Student’s *t*-test was used for normally distributed data. Weekly changes in symptom frequency in treatment groups were analyzed via Cochran’s *Q* test. Wilcoxon signed-rank test was applied to non-normally distributed paired variables. Data are expressed as mean (standard deviation, SD) or median (min-max), as appropriate. *P *< .05 was considered statistically significant. Post hoc power analysis revealed the statistical power to be 98.3% (effect of weight: *w* = 0.327; alpha = 0.05).

## Results

### Patient Demographics and Treatment Compliance

Overall, the mean (SD) patient age was 50.9 (9.9 years; range 31–80 years), and females composed 74.4% of the study population. There was a significantly higher percentage of females in the MET group than in the MET-PRO group (83.3% vs 63.9%, *P *= .006) (Table 1).

MET-PRO therapy was associated with a significantly higher rate of treatment compliance than MET therapy (91.7% vs 71.4%, *P *= .001). Of 30 patients non-compliant with the treatment, 24 (80.0%) were metformin-treated patients, while only 6 (20.0%) were under MET-PRO therapy (*P *= .001). Patients who were compliant (n = 126) and non-compliant (n = 30) with treatment were similar in terms of age (50.9 [9.7] vs 50.4 [10.8] years, *P *= .820) and gender (females: 92/126 [73.0%] vs 24/30 [80.0%], *P *= .431).

Overall 24 (28.6%) patients in the MET group and 6 (8.3%) patients in the MET-PRO group were considered to be non-compliant. Bloating was a more common cause of non-compliance (19.1% vs 2.8%, *P* = .002) and the development of a later non-compliance (at least 15 days after the therapy) was more likely in the MET versus MET-PRO group (*P* = .002-.001) (Table 1).

### Laboratory Findings in Patients Compliant with Treatment

Median HbA1c levels significantly decreased from baseline to the third month of therapy in both MET (from 6.4% to 5.9%, *P *< .001) and MET-PRO (from 6.4% to 5.6%, *P *< .001) groups, whereas HbA1c values at the third month of therapy were significantly lower in the MET-PRO versus MET group (*P *< .001) with a greater reduction from baseline values (0.9 [0.4-1.6]% vs 0.4 [0-1.6]%, *P *< .001) in this group (Table 2).

At the third month of therapy, a significant reduction from baseline FBG levels (from median 108.0 mg/dL to 106.0 mg/dL, *P *= .003) was noted only in the MET-PRO group, while a significant increase from baseline HDL-C (median 55.5 mg/dL vs 54.5 mg/dL, *P *= .025) and reduction from baseline body weight (85.5-81.0 kg, *P *< .001) and BMI levels (33.6-31.6 kg/m^2^, *P *< .001) were noted only in the MET group (Table 2).

MET-PRO therapy was also associated with a significant increase in median LDL-C levels from baseline to the third month (123.0-154.0 mg/dL, *P *= .017) (Table 2).

### Weekly Symptom Frequency in Patients Compliant with Treatment

**Figure 1.**
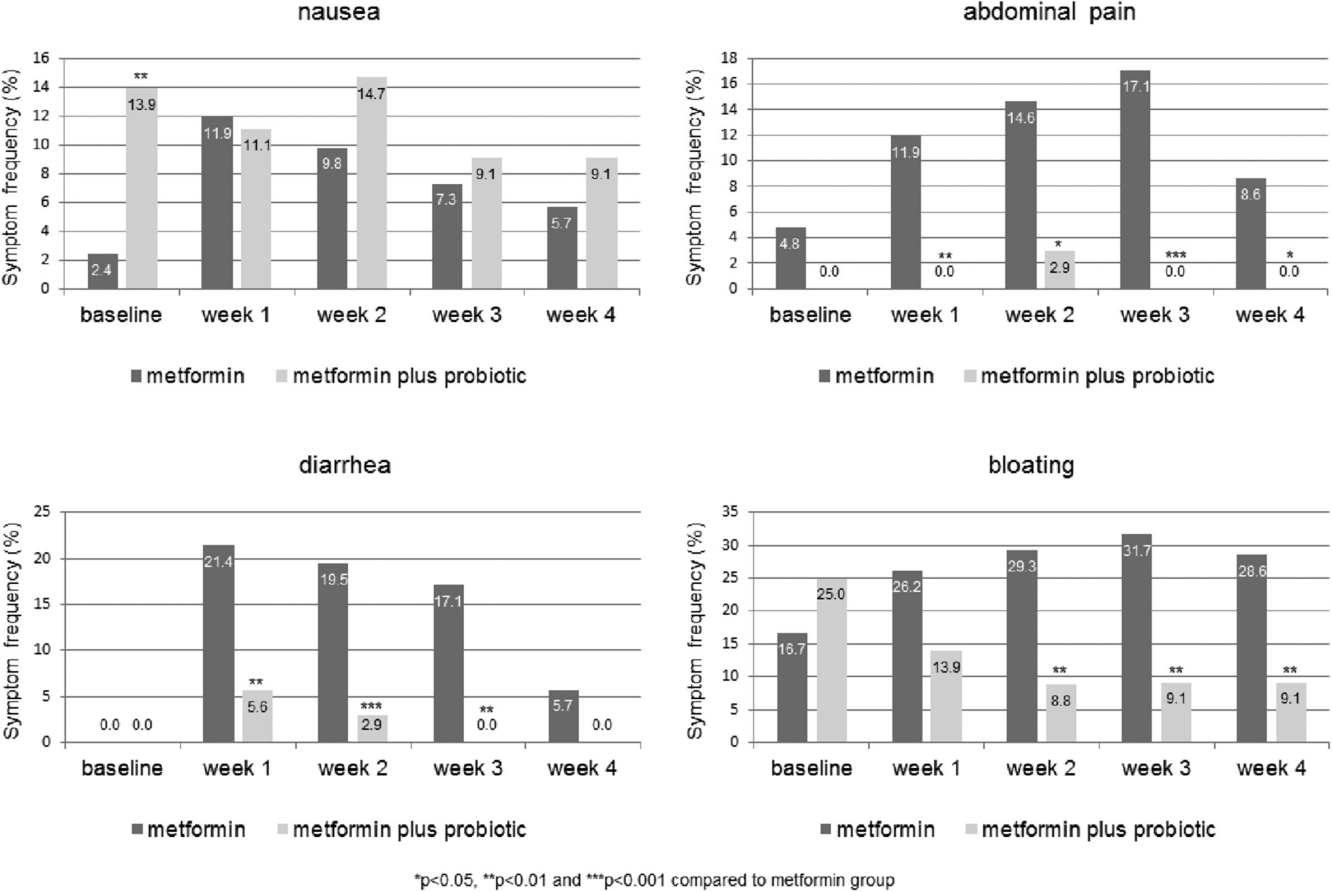
Weekly gastrointestinal symptom frequency in metformin alone and metformin plus probiotic (BB-12) groups of patients with treatment compliance.

At baseline, nausea (13.9% vs 2.4%, *P *= .007) was more common in the MET-PRO group. MET versus MET-PRO therapy was associated with a significantly higher likelihood of abdominal pain (11.9% vs 0.0%, *P* = .002 at week 1, 8.6% vs 0.0%, *P* = .028 at week 4), diarrhea (21.4% vs 5.6%, *P* = .005 at week 1, 17.1% vs 0.0%, *P* < .001 at week 3), and bloating (29.3% vs 8.8%, *P* = .002 at week 2, 28.6% vs 9.1%, *P* = .004 at week 4) (Table 3, Figure 1).

## Discussion

Our findings revealed the concomitant use of probiotic (BB-12) in metformin-treated patients with T2D or prediabetes to enable the reduced risk of metformin-related gastrointestinal side effects, a higher rate of compliance with metformin therapy, and a greater reduction in HbA1c levels.

*B. lactis* is considered among the potential probiotics that can influence glucose homeostasis and insulin resistance by increasing glycogen synthesis and decreasing the expression of hepatic gluconeogenesis-related genes, as well as by improvement of the translocation of glucose transporter-4 (GLUT4) and insulin-stimulated glucose uptake.^7,15^ In a systematic review of nine randomized controlled trials with adult T2D patients regarding the effects of probiotics on glycaemic outcomes, authors noted that the multistrain probiotics that contain *Lactobacillus acidophilus, Streptococcus thermophilus, Lactobacillus bulgaricus*, and/or *Bifidobacterium lactis* administered for 6-12 weeks can be efficacious for improving glycemic control in T2D.^16^ The use of a multi-strain probiotic supplementation (UB0316; *Lactobacillus, Bifidobacterium*, and *Bacillus* strains) versus placebo for 12-weeks in 79 T2D patients was reported to be associated with significantly improved glycemic control by a greater reduction in HbA1c and significantly reduced body weight, but with similar changes in FBG, HOMA-IR, insulin, and HDL-C and LDL-C levels.^17^

In addition, *B. animalis* ssp. *lactis* 420 (B420) was reported to improve insulin sensitivity and glucose tolerance and to decrease fat mass in dietary mouse models of diabetes and obesity,^18^ while the combined use of B420 with sitagliptin or metformin in diabetic mice was reported to be effective in reducing glycemic response and plasma glucose concentration.^11^ In other studies of experimental diabetes models, metformin plus prebiotic use was also reported to reduce hyperglycemia and adiposity and to improve FBG, glucose tolerance, and insulin resistance, as compared with metformin alone.^7,19,20^

Notably, both the therapeutic and undesired digestive effects of metformin are suggested to be mediated through alterations in gut microbiota including enrichment of SCFA, butyrate, and propionate, producing microbiota as well as an increase in abundance of *Escherichia* species.^9-11^ In addition, probiotics were reported to be associated with an increase in the levels of these SCFA-producing bacteria (mainly *Lactobacillus* and *Bifidobacterium*) and a decrease in the levels of *Escherichia* and thereby improving the gut-barrier function and promoting the GLP-1 and GLP-1-induced insulin secretions resulting in effectively improved blood glucose and blood lipid parameters.^21-23^

The beneficial effects of probiotic BB-12 in improved glycemic control in our metformin-treated patients seem notable in this regard, given that BB-12 provides not only a source of SCFA-producing bacteria that favor the therapeutic effects of metformin but also enables higher compliance with metformin treatment with increased likelihood of treatment adherence and usage of therapeutic doses of the drug via preventing the gastrointestinal side effects of metformin. Accordingly, the superiority of metformin plus BB-12 over metformin alone in improved glycemic control in the current study seems to be based on both direct (microbiota-mediated glucose homeostasis) and indirect (increased compliance to metformin therapy) effects of BB-12 on glucose homeostasis. Hence, our findings support that targeting the gut microbiota could offer an alternative therapeutic approach that may also consider the substantial inter-individual variability in the management of T2D.^2,3^

Additionally, metformin-induced changes in microbiota are proposed to result not only in the glucose homeostasis but also in hepatic glucose production-mediated appetite suppression, which can contribute to weight reduction and glycemic control.^24,25^ Diabetes Prevention Study (DPP) revealed the weight loss associated with metformin to be sustained and safe and to be strongly dependent on the adherence rate of the participants.^26^ The significant reduction obtained in body weight and BMI in metformin-treated patients in the current study seems to be consistent with these reports. However, despite significantly higher adherence to metformin treatment with the combined use of probiotic in the current study, a significant reduction from baseline in body weight and BMI was noted only in the metformin group along with a significant increase from baseline in LDL-C only in the metformin plus probiotic group.

Nonetheless, it should be noted that while changes in microbiota play a functional role in the beneficial metabolic effects of metformin, the extent to which changes in microbiota are necessary for the metformin impact remains unknown.^4,27^ Notably, in a recent study addressing this issue, the authors reported that the ability of metformin to beneficially impact metabolic syndrome in mice was not impacted by ablation of gut microbiota achieved by the use of antibiotics or germ-free mice and that obesity, hepatic steatosis, and low-grade inflammation were similarly suppressed by metformin in the presence or absence of gut microbiota.^28^

The current evidence suggests that the weight change associated with metformin is more likely to be due to decreased caloric intake and appetite regulation both directly and indirectly through its gastrointestinal side effects.^27^ In fact, the extent of weight loss with metformin therapy and its relationship to gastrointestinal side effects remains unclear.^29^ Given that no reduction in body weight was noted in our metformin plus probiotic group, prevention of gastrointestinal side effects via probiotic in this group may have also resulted in a decrease in the weight-loss effect of metformin. Hence, our findings emphasize the findings from the DPP trial, which has suggested that adherence to metformin therapy correlates with weight loss, but whether gastrointestinal side effects are a harbinger of metformin’s efficacy for weight loss is still inconclusive.^29^

The meta-analyses on the efficacy of probiotics for weight loss revealed inconsistent findings. In 2 meta-analyses, the authors concluded that probiotics were ineffective in controlling weight changes,^30,31^ while in another meta-analysis, probiotic use was concluded to be associated with a significant reduction in body weight, particularly when used for a longer duration.^32^ In a past study, metformin therapy (300 mg/kg/day) was reported to be associated with decreased* Bifidobacterium* spp. in the gut microbiota of obese rats and authors emphasized evaluation of not only the dietary factors known to alter gut microbiota but also the effects of pharmacological agents such as metformin used to treat critical metabolic diseases.^33^

Nonetheless, it should be noted that the use of probiotics to prevent and treat obesity and related problems remains debated with limited data on in-depth microbial evaluations to indicate which bacterial genera and/or species are strictly related to weight modulation and obesity development.^34^ Accordingly, the probiotic effect on body weight and metabolism has been suggested to be strain-specific with the efficacy of only some of the species included in the* Lactobacillus* and *Bifidobacterium* genera and the likelihood of deleterious outcomes with the use of other strains.^34^

In a past study with 360 metformin-treated T2D patients, diarrhea (21.1%), followed by heartburn (52.1%), nausea (47.4%), abdominal pain (35.5%), bloating (35.2%), and retching (21.1%) were indicated to be the most commonly reported gastrointestinal symptoms, while bloating, nausea, and abdominal pain were determined to be significantly associated with patient nonadherence or physician reluctance to optimally titrate the metformin dose.^35^ In another study with 361 newly diagnosed patients who were randomly assigned to 1000 mg/day, 1500 mg/day, and 2000 mg/day doses of metformin, authors reported a dose-dependent increase in glycemic control along with similar rates of gastrointestinal side effects resulting in discontinuation of treatment by 17.2% of patients within the first 4 weeks in most of cases, regardless of the metformin dosage.^36^

Metformin-related gastrointestinal side effects, albeit reported by a lower percentage of patients possibly in relation to the inclusion of previously treatment-naïve patients without IBD or autonomous neuropathy in the current study, resulted in the experience of non-compliance with treatment by 24 (28.6%) patients who received metformin without probiotic BB-12. Gastrointestinal intolerance symptoms, bloating, in particular, were the leading cause of treatment non-compliance in our metformin-treated patients, especially after 15 days of treatment with the introduction of higher doses of metformin. Likewise, in a past study among T2D patients with metformin intolerance, the use of a gastrointestinal microbiome modulator (GIMM) in combination with metformin was reported to ameliorate the gastrointestinal symptoms along with a significantly greater reduction in FBG in the metformin-GIMM combination group than in the metformin-placebo group.^37^ The authors noted that combining a GIMM with metformin might allow the greater use of metformin and improve the management of disease in patients with T2D.^37^

In fact, the gastrointestinal side effects of metformin are suggested to be associated with the adverse impact of metformin on folate-producing gut microbiota as well as on folic acid absorption and thus folate-producing probiotics such as *Bifidobacteria* have been considered to help with gastrointestinal side effects of metformin.^38^

Certain limitations to this study should be considered. First, the potential lack of generalizability is an important limitation due to the single-center study design with relatively small sample size. Second, given the potential psychological effects of probiotic support, the lack of a placebo group seems to be another limitation of the present study. Third, the assessment of the weekly symptom frequency was based on subjective reporting along with a lack of items on symptom severity and weekly change in symptom severity in the questionnaire. Nevertheless, despite these certain limitations, given the restricted amount of data on patients treated with probiotic plus anti-diabetic medication in real-life practice, providing data on a detailed assessment of treatment compliance based on not only the treatment discontinuation but also on the dose skipping and dose reduction parameters, our findings represent a valuable contribution to the literature.

In conclusion, metformin treatment is the most important step in T2DM treatment and our most important problem in metformin treatment is non-compliance with treatment due to gastrointestinal system side effects. In our study, probiotic support added to metformin treatment decreased the complaints of diarrhea and bloating and increased the compliance of the patients to the treatment. Better glycemic control and better HbA1c reduction were observed in patients receiving probiotic supplementation, with increased adherence to the treatment and possible effects of probiotics on the intestinal-pancreatic axis. In summary, in patients who added probiotic support to metformin treatment, side effects decreased, and better adherence to treatment and better HbA1c reduction were observed. Questioning our results in similar studies and supporting them with meta-analyses is extremely important in terms of adding probiotic support to metformin treatment for clinical use.

## Figures and Tables

**Table 1.** Patient Demographics and Compliance with Treatment

	**Total (n = 156)**	**MET (n = 84)**	**MET-PRO (n = 72)**	***P***
**Age (years), mean (SD, min-max)**	50.9 ± 9.9 (31-80)	50.4 ± 9.4 (31-68)	51.2 ± 10.4 (32-80)	.597
**Gender, n (%)**				
Female	116 (74.4)	70 (83.3)	46 (63.9)	**.006**
Male	40 (25.6)	14 (16.7)	26 (36.1)	
**Treatment compliance, n (%)**				
Compliant with treatment	126 (80.8)	60 (71.4)	66 (91.7)	**.001**
Non-compliant with treatment	30 (19.2)	24 (28.6)	6 (8.3)	
**Reason for non-compliance, n (%)**				
Nausea	2 (6.7)	0 (0)	2 (2.8)	.211
Diarrhea	10 (33.3)	8 (9.5)	2 (2.8)	.108
Bloating	18 (60.0)	16 (19.1)	2 (2.8)	**.002**
**Time of non-compliance, n (%)**				
0-7 days	6 (20.0)	2 (2.4)	4 (5.6)	.416
8-14 days	2 (6.7)	0 (0.0)	2 (2.8)	.211
15-21 days	12 (40.0)	12 (14.3)	0 (0.0)	**.001**
22-28 days	10 (33.3)	10 (11.9)	0 (0.0)	**.002**

MET, metformin alone; MET-PRO, metformin plus probiotic.

Student’s *t* test, Pearson χ^2^ test, Fisher’s exact test.

**Table 2.** Laboratory Findings in Patients Compliant with Treatment (n = 126)

	**Total (n = 126)**	**MET (n = 60)**	**MET-PRO (n = 66)**	***P***
**Laboratory findings, median (min-max)**				
**HbA1c (%)**				
Baseline	6.4 (5.5-9.9)	6.4 (5.9-9.9)	6.4 (5.5-7.4)	.791
Third month	5.6 (4.9-8.4)	5.9 (5.2-8.4)	5.6 (4.9-6.2)	**<.001**
*P*	**<.001**	**<.001**	**<.001**	
Change from baseline	0.7 (0-1.6)	0.4 (0-1.6)	0.9 (0.4-1.6)	**<.001**
**FBG (mg/dL)**				
Baseline	107 (82-285)	102.5 (82-285)	108 (83-196)	.052
Third month	105 (68-158)	104.5 (68-158)	106 (82-129)	.725
*P*	**.009**	.508	**.003**	
Change from baseline	3 (−32 to 127)	2 (−32 to 127)	6 (−20 to 114)	.107
**HDL-C (mg/dL)**				
Baseline	54 (26-87)	54.5 (26-69)	53 (32-87)	.769
Third month	53 (33-85)	55.5 (33-76)	50 (33-85)	**.028**
*P*	.533	**.025**	.361	
Change from baseline	−1 (−29 to 33)	−2 (−15 to 16)	1 (−29 to 33)	**.038**
**LDL-C (mg/dL)**				
Baseline	133 (82-249)	146.5 (97-249)	123 (82-236)	.140
Third month	154 (86-239)	150 (86-228)	154 (93-239)	.938
*P*	**.012**	.263	**.017**	
Change from baseline	−12 (−133 to 154)	−10 (−109 to 154)	−13 (−133 to 70)	.197
**Weight (kg)**				
Baseline	84 (47-132)	85.5 (55-120)	80 (47-132)	**.007**
Third month	81 (53-127)	81 (53-127)	81 (57-127)	.120
*P*	**<.001**	**<.001**	.386	
Change from baseline	2.83 (−78.72 to 29.55)	3.64 (−47.67 to 14.44)	1.15 (−78.72 to 29.55)	**.008**
**BMI (kg/m ^2^ )**				
Baseline	30.8 (19.3-48.3)	33.6 (21.5-44.6)	29.1 (19.3-48.3)	**<.001**
Third month	30 (20.7-48.3)	31.6 (20.7-44)	29.1 (22.2-48.3)	**.009**
*P*	**<.001**	**<.001**	.254	
Change from baseline	2.56 (−93.26 to 31.27)	3.04 (−15.12 to 14.58)	1.9 (−93.26 to 31.27)	.087

MET, metformin alone; MET-PRO, metformin plus probiotic; FBG, fasting blood glucose; HDL-C, high-density lipoprotein cholesterol; LDL-C, low-density lipoprotein cholesterol; BMI, body mass index.

Mann–Whitney *U* test, Wilcoxon signed-rank test.

**Table 3.** Distribution of Weekly Symptom Frequency in MET Versus MET-PRO Groups of Patients with Treatment Compliance

	**Baseline**			**Week 1**			**Week 2**			**Week 3**			**Week 4**			**Change Over Time**	
	**MET** **(n = 84)**	**MET-P** **(n = 72)**	***P***	**MET** **(n = 82)**	**MET-P (n = 68)**	***P***	**MET** **(n = 82)**	**MET-P** **(n = 66)**	***P***	**MET** **(n = 70)**	**MET-P** **(n = 66)**	***P***	**MET** **(n = 60)**	**MET-P** **(n = 66)**	***P***	**MET**	**MET-P**
**Symptoms, n (%)**																	
Loss of appetite	0 (0.0)	2 (2.8)	.211	2 (2.4)	0 (0)	.500	0 (0)	0 (0)	-	0 (0)	0 (0)	-	0 (0)	0 (0)	-	0.092	0.092
Nausea	2 (2.4)	10 (13.9)	**.007**	10 (11.9)	8 (11.1)	.877	8 (9.8)	10 (14.7)	.353	6 (7.3)	6 (9.1)	.694	4 (5.7)	6 (9.1)	0.523	0.136	0.366
Vomiting	0 (0.0)	0 (0.0)	-	2 (2.4)	0 (0.0)	.500	2 (2.4)	2 (2.9)	.999	2 (2.4)	0 (0)	.502	2 (2.9)	0 (0)	0.497	0.525	-
Taste disturbance	2 (2.4)	2 (2.8)	.999	6 (7.1)	0 (0.0)	**.031**	4 (4.9)	0 (0)	.127	4 (4.9)	0 (0)	.129	2 (2.9)	0 (0)	0.497	**0.040**	0.092
Dizziness	4 (4.8)	2 (2.8)	.687	2 (2.4)	2 (2.8)	.999	4 (4.9)	4 (5.9)	.999	4 (4.9)	4 (6.1)	.999	4 (5.7)	4 (6.1)	0.999	0.525	0.092
Abdominal pain	4 (4.8)	0 (0.0)	.125	10 (11.9)	0 (0.0)	**.002**	12 (14.6)	2 (2.9)	**.014**	14 (17.1)	0 (0)	**<.001**	6 (8.6)	0 (0)	**0.028**	0.058	-
Headache	14 (16.7)	4 (5.6)	**.030**	6 (7.1)	4 (5.6)	.753	6 (7.3)	0 (0)	**.032**	6 (7.3)	0 (0)	**.033**	6 (8.6)	0 (0)	**0.028**	**<0.001**	**0.003**
Diarrhea	0 (0.0)	0 (0.0)	-	18 (21.4)	4 (5.6)	**.005**	16 (19.5)	2 (2.9)	**.002**	14 (17.1)	0 (0)	**<.001**	4 (5.7)	0 (0)	0.120	**0.002**	0.092
Bloating	14 (16.7)	18 (25.0)	.199	22 (26.2)	10 (13.9)	.058	24 (29.3)	6 (8.8)	**.002**	26 (31.7)	6 (9.1)	**.001**	20 (28.6)	6 (9.1)	**0.004**	**0.009**	**<0.001**

MET, metformin alone; MET-P, metformin plus probiotic Pearson chi-square test, Fisher’s exact test, Cochran’s *Q* test.
